# Selections

**Published:** 1896-11

**Authors:** 


### Selections.

### THE VALUE OF ANTIPYRETICS.

Binz, of Bonn, discussed the relative value of quinine, salicylic acid, antipyrin, antifebrin, phenacetin, thallin, and alcohol. He stated that until 1867 quinine had universally passed as a drug acting in malaria through its effect on the nervous system. His researches at that time had demonstrated, however, that it was a powerful poison for protoplasm of a lower order, and especially that produced in media in which decomposition of vegetable substances takes place. His experiments had also demonstrated that the antipyretic action of quinine was generally independent of the nervous centres and the circulation, and that the drug exercised a paralyzant action on an inferior organism which was the cause of malarial fever. The discovery of Laveran and the researches of his successors have since shown the exactitude of these assertions. The lowering of febrile temperature in other diseases, as well as in healthy, warm-blooded animals, is due to the fact that the activity of the disassimilating cells is directly affected. This fact is demonstrated by experimental evidence (1) that quinine diminishes the number and vitality of the leucocytes; (2) that it diminishes the quantity of nitrogen and sulphur in the urine of warm-blooded animals, whether healthy or suffering from fever; (3) that it lowers their internal temperature when they are submitted to the action of a hot vapor-bath; (4) that in Rubner's calorimeter (which enables one to study the subject of the experiment and the control animal at the same time, and find absolutely similar conditions) under the influence of quinine there is a decreased production of vapor in warm-blooded animals, whether healthy or febrile. Quinine is an antipyretic through the influence which it exercises both on the pathogenic cells which cause malaria and on the cells which form a normal portion of the organism. It has thus a special as well as a general antipyretic influence.—*University Medical Journal.*

